# Spontaneous community-acquired PVL-producing *Staphylococcus aureus* mediastinitis in an immunocompetent adult – a case report

**DOI:** 10.1186/s12879-020-05076-6

**Published:** 2020-05-19

**Authors:** Josselin Brisset, Thomas Daix, Jérémy Tricard, Bruno Evrard, Philippe Vignon, Olivier Barraud, Bruno François

**Affiliations:** 1 Réanimation polyvalente, CHU Dupuytren, 2 avenue Martin Luther King, F-87000 Limoges, France; 2 Maladies infectieuses, CHU Dupuytren, F-87000 Limoges, France; 3 Inserm CIC 1435 & UMR 1092, CHU Dupuytren, F-87000 Limoges, France; 4 Chirurgie cardiaque, CHU Dupuytren, F-87000 Limoges, France; 5 Laboratoire de Bactériologie - Virologie – Hygiène, CHU Dupuytren, F-87000 Limoges, France

**Keywords:** Community-acquired mediastinitis, MSSA, Panton-valentine Leucocidin

## Abstract

**Background:**

Mediastinitis caused by hematogenous spread of an infection is rare. We report the first known case of community-acquired mediastinitis from hematogenous origin in an immunocompetent adult. This rare invasive infection was due to Panton-Valentine Leucocidin-producing (PVL+) methicillin-susceptible *Staphylococcus aureus* (MSSA).

**Case presentation:**

A 22-year-old obese man without other medical history was hospitalized for febrile precordial chest pain. He reported a cutaneous back abscess 3 weeks before. CT-scan was consistent with mediastinitis and blood cultures grew for a PVL+ MSSA. Intravenous clindamycin (600 mg t.i.d) and cloxacillin (2 g q.i.d.), secondary changed for fosfomycin (4 g q.i.d.) because of a related toxidermia, was administered. Surgical drainage was performed and confirmed the presence of a mediastinal abscess associated with a fistula between the mediastinum and right pleural space. All local bacteriological samples also grew for PVL+ MSSA. In addition to clindamycin, intravenous fosfomycin was switched to trimethoprim-sulfamethoxazole after 4 weeks for a total of 10 weeks of antibiotics.

**Conclusions:**

We present the first community-acquired mediastinitis of hematogenous origin with PVL+ MSSA. Clinical evolution was favorable after surgical drainage and 10 weeks of antibiotics. The specific virulence of MSSA PVL+ strains played presumably a key role in this rare invasive clinical presentation.

## Background

Mediastinitis is mainly due to deep sternal wound infection, esophageal perforation, or descending necrotizing mediastinitis originating from ear-nose-throat (ENT) infections and exceptionally to hematogenous spread [1]. In case of hematogenous spread, mediastinitis is usually healthcare-acquired [2, 3].

Panton Valentin Leucocidin-producing (PVL+) methicillin-susceptible *Staphylococcus aureus* (MSSA) was mostly described in community-acquired necrotizing pneumonia, bone and joint infections and skin and soft tissue infections such as furunculosis [4]. As far as we know, we present the first case of a community-acquired mediastinitis caused by MSSA. The strain was PVL+ and seemed to belong to USA300 strains [5] which are increasingly associated with invasive infections.

## Case presentation

A 22-year-old obese (BMI = 38 kg/m^2^) man without other medical history was admitted to the emergency department (ED) for precordial chest pain worsening for 5 days and radiating to the back and shoulders. The patient had low dysphagia, progressive-onset dyspnea and unproductive cough for 2 days but without fever or shiver. This patient, usually living in Illinois, had worked as a teacher in France for the last 8 months and did not travel outside Western Europe and the USA.

On admission, the patient presented with fever (38.5 °C), tachypnea (RR: 30/min) and required 3 l/min of oxygen (SpO_2_: 97%) but had no signs of respiratory distress. Lung auscultation revealed decreased breath sounds in the right lower lobe. Blood pressure and heart rate were normal. There was no evidence for a dental, oro-pharyngeal infection, or cervical cellulitis. Second questioning of the patient highlighted a skin lesion described as an abscess in the back 3 weeks before admission which was successfully treated by Povidone-Iodine alcohol but it was absent on the current clinical examination. None of his relatives or colleagues described any signs of skin infection.

**Fig. 1:**
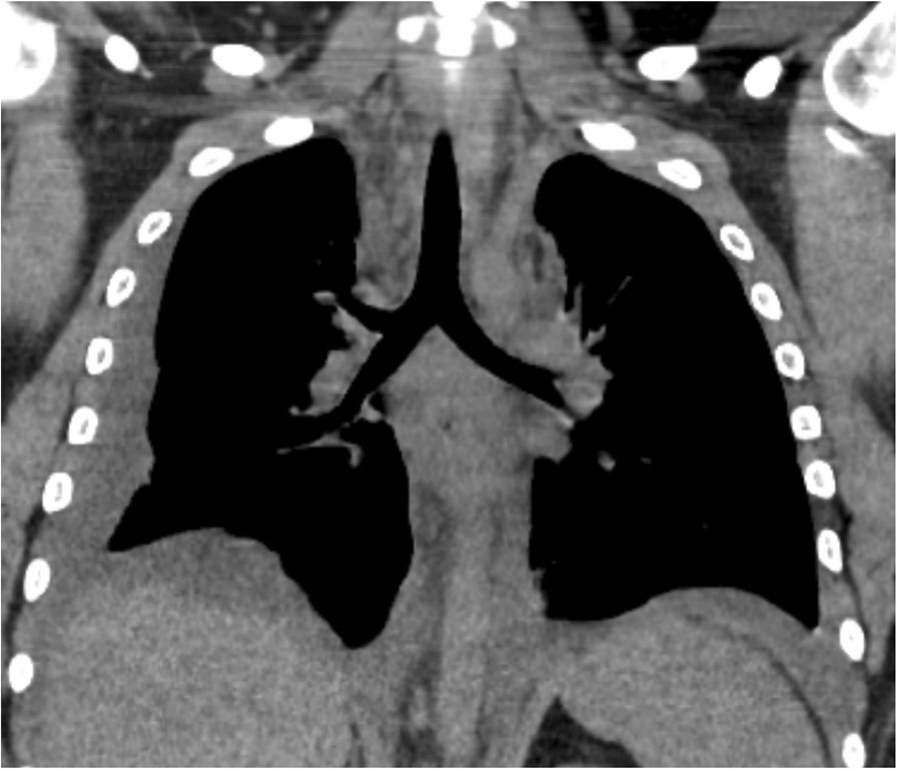
Chest CT scan of the initial presentation of the mediastinitis, with diffuse mediastinal infiltration

Blood tests were consistent with a marked inflammatory syndrome with a high leucocytes level (41,000 /mm^3^; 82% of neutrophils) and a CRP of 450 mg/l. Procalcitonin was 3.3 ng/ml and lactate was 1.31 mmol/l. Neck and chest CT-scan revealed an enlargement of the mediastinum due to a diffuse mediastinal infiltration with a moderate bilateral pleural effusion (Fig. 1), without lung parenchymal, pharyngeal and neck abnormalities or jugular venous thrombosis. Esophageal perforation was ruled out by a Barium swallow test and esophagogastroduodenoscopy. Community-acquired mediastinitis being suspected, empirical antibiotic treatment with intravenous amoxicillin/clavulanic acid 1 g q.i.d. was started in the ED and the patient was transferred in the intensive care unit (ICU).

On ICU admission, right pleural tap evidenced purulent fluid with Gram positive cocci. Cultures grew for a PVL+ MSSA. The genomic analysis revealed that the *Staphylococcus aureus* strain belonged to a CC8 clonal complex. In addition to PVL, the strain exhibited enterotoxins K, Q, and an *agr*1 allele (Identibac *S. aureus* Genotyping DNA microarray, Alere Technologies, Jena, Germany). Blood cultures sampled at ICU admission were also positive for the same PVL+ MSSA.

Transthoracic and transesophageal echocardiography ruled out infective endocarditis. On day 2, the antibiotic regimen was switched to intravenous cloxacillin (2 g q.i.d.) and clindamycin (600 mg t.i.d.) as an anti-toxinic PVL adjunctive treatment. On day 5, a diffuse skin rash consistent with a toxidermia appeared and lead to the replacement of cloxacillin with fosfomycin (4 g q.i.d.) with no skin rash recurrence.

**Fig. 2:**
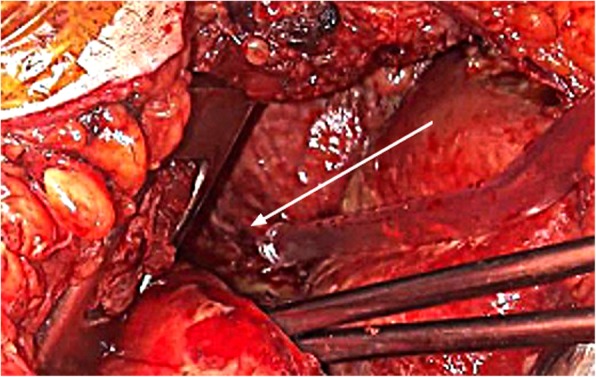
This picture taken during surgery, evidenced the fistula (white arrow) from the mediastinum to the right pleura

On day 4, cervicotomy and right thoracotomy allowed the evacuation of a right pleural empyema and mediastinal abscesses, and evidenced the presence of a fistula between the mediastinum and right pleural space (Fig. 2). All surgical samples from the mediastinum and the right pleura grew for the same MSSA strain.

**Fig. 3:**
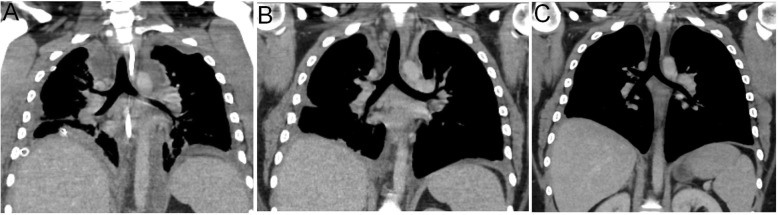
(**a**) Day 7 CT scan- acme of the mediastinal infiltration, with abscesses formation (3 days after surgery); (**b**) CT scan at M1 after intensive care unit admission; (**c**) CT scan at M2 after intensive care unit admission

Blood cultures obtained systematically on days 8, 15 and 22 were negative. Subsequent echocardiography and chest CT-scan depicted the regression of both the mediastinal infiltration and residual abscesses (Fig. 3a and b). The patient was discharged from ICU on day 36 and from hospital on day 42 without any complication. Chest CT-scan performed two months later confirmed the complete disappearance of mediastinal abscesses (Fig. 3c).

On top of clindamycin, intravenous fosfomycin was carried on for 4 weeks, then switched to trimethoprim-sulfamethoxazole until hospital discharge (day 42). Clindamycin and trimethoprim-sulfamethoxazole were continued for 6 more weeks for a total of 10 weeks of treatment.

No immunodepression was evidenced with normal immunoglobulin levels, no complement pathway defect, negative HIV testing and antibody titers of previously administered vaccines (diphtheria, tetanus, HBV) without any abnormal response.

Long-term antibiotics and early surgery benefited our patient which is now doing well and was able to go back to the United States of America (USA) on June 6th, 2019.

## Discussion and conclusions

The MSSA strain was PVL+ and a member of CC8 clonal complex. Regarding the genetic marker interference methodology, this strain belongs to USA300 strains [5]. The prevalence of this strain seems to increase and to be associated with invasive infection [6].

Undoubtedly, the PVL toxin may have significantly contributed to the severity of the disease. PVL is expressed by major methicillin-resistant *Staphylococcus aureus* (MRSA) clones, which have now spread throughout the world [7] even if PVL+ SA remains especially present in the USA, with 48.1% of MRSA strains and 11.5% of MSSA strains being identified in this region (8.2% in northeast, region of origin of our patient) [8]. In contrast in France, the majority of strains expressing PVL are MSSA strains. As in our patient, PVL+ SA strains affect mainly young adults without underlying disorder [9] and are more often found in community-acquired infections than in hospital-acquired infections [10]. In community-acquired infections, PVL is an independent marker of severity regardless the resistance of the *Staphylococcus aureus* strain to methicillin [11]*.* PVL is known to lyse neutrophils and boost the inflammatory cascade which can explain the progressive tissue necrosis in case of invasive infection associated with necrotic skin and soft tissue infection such as furuncles [12] and community-acquired pneumonia [13]. In our patient, these properties presumably contributed to the development of a fistula from which facilitated the spread of the local infection from the mediastinum to the right pleural space. PVL+ SA toxins have been most frequently associated with skin and soft tissue infections (folliculitis and cutaneous abscesses), and with necrotizing pneumonia [4]. Since the medical history, clinical examination and CT-scan did not show any signs of dental, pharyngeal or cervical infections, we can assume that the skin abscess which developed 3 weeks before the severe infection could be the source of the initial bacteremia and secondary location in the chest.

Three main etiologies of acute infective mediatinitis are traditionally distinguished: deep sternal wound infection following cardiovascular and thoracic surgery, esophageal perforation and descending necrotizing mediastinitis originating from dental, pharyngeal or cervical infections [14]. Overall mortality of acute mediastinitis ranges between 20% (esophageal perforation) to 30% (descending necrotizing mediastinitis) [1]. Exceptionally, primary mediastinitis may result from a bacteremia. To our knowledge, only two cases have been reported so far. Both of them were healthcare-associated mediastinitis, one in a dialysis patient with an arteriovenous shunt due to MRSA which was not identified as PVL+ [2] and one associated with a central venous catheter and due to *Candida* species [3].

Treatment of community-acquired acute mediastinitis is based on antimicrobial therapy and control of the source of infection using surgical debridement. With the exception of mediastinitis developing in the postoperative setting which requires rapid surgical debridement within 24 h [15], there are currently no guidelines for the timing of the surgery. Multiple surgical management is required in approximatively 30% of patients [16]. There are currently no guidelines for empirical antibiotic therapy which should be adapted on the expected origin of mediastinitis. With the exception of deep sternal wound infections which are commonly related to SA, polymicrobial infection with flora from the oral cavity and/or the upper respiratory tract is common in other causes of mediastinitis [1]. In our patient, the empirical therapy with amoxicillin-clavulanic acid targeted aerobic and anaerobic oral and upper gastro-intestinal bacteria. Antibiotic regimen was adapted secondary to MSSA identification using high-dose of cloxacillin since beta-lactam antibiotics can increase PVL secretion in case of insufficient concentration in vitro [17]. In addition, clindamycin was associated since it strongly inhibits PVL secretion in vitro, as linezolid [17].

PVL-producing MSSA can result in community-acquired infective mediastinitis secondary to bacteremia after a cutaneous abscess in young immunocompetent adult.

## Data Availability

Data sharing is not applicable to this article as no datasets were generated or analyzed during the current case.

## Abbreviations

BMI: Body mass index
CRP: C-reactive protein
ED: Emergency department
HBV: Hepatitis B virus
HIV: Human immunodeficiency virus
ICU: Intensive care unit
MRSA: Methicillin-resistant *Staphylococcus aureus*
MSSA: Methicillin-susceptible *Staphylococcus aureus*
PCT: Procalcitonin
PVL: Panton-Valentine Leucocidin
RR: Respiratory rate
TTE: Transthoracic echocardiography
USA: United States of America
WBC: White blood cells
